# Supplementary material for: Lack of head sparing following third-trimester caloric restriction among Tanzanian Maasai
Source: PLoS One. 2020 Sep 23;15(9):e0237700. doi: 10.1371/journal.pone.0237700 (PMC7510984; doi:10.1371/journal.pone.0237700)
Supplement: S1 Fig — Data collection was centered in Endulen, but subjects were enrolled from within the entire NCA. Adapted from Coast (2001). (DOCX) [file pone.0237700.s001.docx]

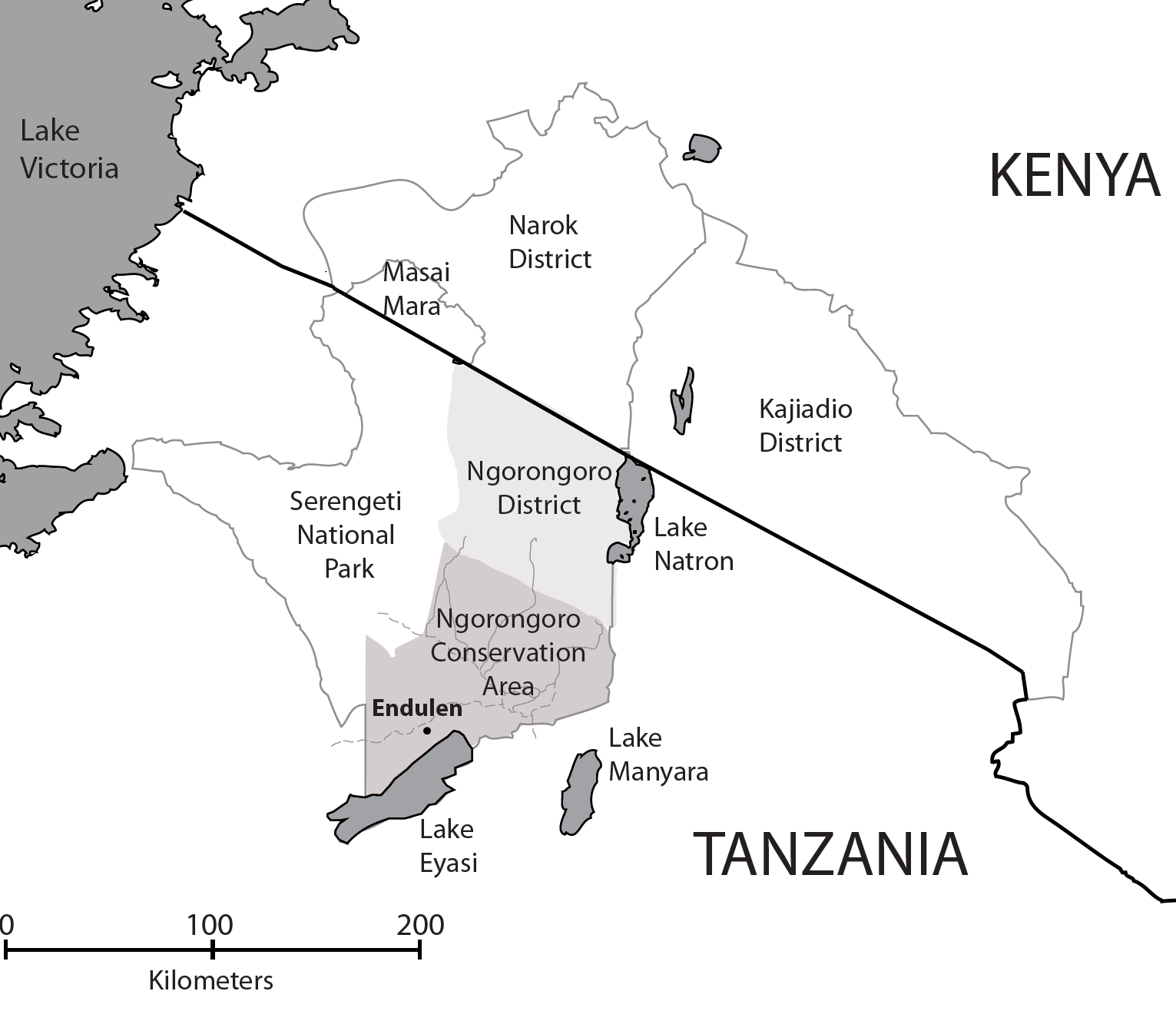
**S1 Fig.** Map of Northern Tanzania and Southern Kenya showing the location of the NCA (dark shaded). Data collection was centered in Endulen, but subjects were enrolled from within the entire NCA. Adapted from Coast (2001).

Coast E. Maasai Demography. London LSE; 2001.
