# Supplementary figures and images for: Lack of head sparing following third-trimester caloric restriction among Tanzanian Maasai
Source: PLoS One. 2020 Sep 23;15(9):e0237700. doi: 10.1371/journal.pone.0237700 (PMC7510984; doi:10.1371/journal.pone.0237700)

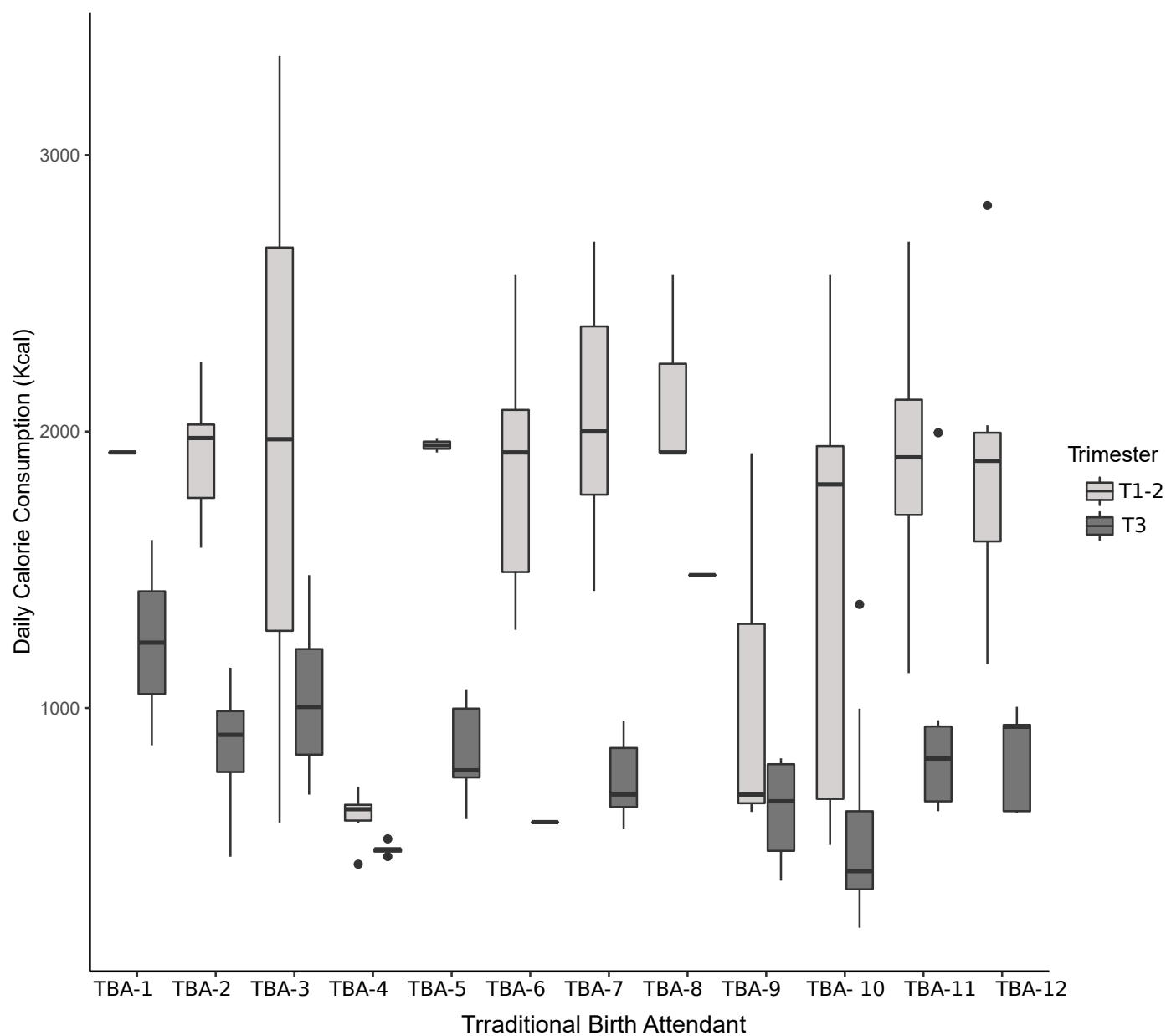

Supplement: S2 Fig — (PDF) [file pone.0237700.s002.pdf]
