# Supplementary material for: Lack of head sparing following third-trimester caloric restriction among Tanzanian Maasai
Source: PLoS One. 2020 Sep 23;15(9):e0237700. doi: 10.1371/journal.pone.0237700 (PMC7510984; doi:10.1371/journal.pone.0237700)
Supplement: S1 Table — (DOCX) [file pone.0237700.s003.docx]

S5 Table. Results of linear mixed models with birth weight (BW) and head circumference (HC) as independent variables, the difference in diet components between early-mid and late pregnancy as dependent variable, and TBA and mother as random factors.

| Anthropometric measurement | Diet component | Estimate | Std. Error | t | p |
| --- | --- | --- | --- | --- | --- |
| BW | Calories | 0.3253 | 0.1391 | 2.338 | **0.0244** |
|  | Protein | 10.71 | 3.61 | 2.968 | **0.00499** |
|  | Fat | 5.272 | 4.468 | 1.18 | 0.245 |
|  | Carbohydrate | 1.0573 | 0.7833 | 1.35 | 0.184 |
| HC | Calories | -0.0002559 | 0.0005554 | -0.461 | 0.647 |
|  | Protein | 0.01011 | 0.01522 | 0.664 | 0.51 |
|  | Fat | -0.006503 | 0.015931 | -0.408 | 0.685 |
|  | Carbohydrate | -0.002914 | 0.002852 | -1.022 | 0.313 |
