# Supplementary material for: Lack of head sparing following third-trimester caloric restriction among Tanzanian Maasai
Source: PLoS One. 2020 Sep 23;15(9):e0237700. doi: 10.1371/journal.pone.0237700 (PMC7510984; doi:10.1371/journal.pone.0237700)

**S1 Appendix.** Data collection booklets

FOOD FREQUENCY QUESTIONNAIRE (FFQ)

The questioner is in English, KiSwahili and KiMaa

EATING & DRINKING

CHAKULA/KINYWAJI

NINYA/NIOK

**Serving sizes.** These serving sizes were based on typical household items, such as standardized measuring cups, used to prepare meals.

X = M (medium) serving size = 1 cup

Y = S (small) is half of M

Z = L (large) is one M plus half of M

BEFORE PREGNANT/ KABLA YA MIMBA/MIRA ENDUA

1 What do you eat/drink in the wet season, when you're not pregnant?

• Unakula nini?/kunywa wakati wa mvua, ukiwa huna mimba?

• kainyoo oshi inya/iok te ngata olari mira endua?

2 How much do you eat/drink each day?

• Unakula kiasi gani/kunywa kila siku?

• ke baa en daa ninya/niok torkekuno?

3 What do you eat/drink in the dry season, when you're not pregnant?

• Unakula nini?/kunywa wakati wa ukame?

• kainyoo oshi inya/iok te ngata olamey mira endua?

4 How much do you eat/drink each day?

• Unakula/kunywa kiasi gani kwa siku?

• ke baa en daa ninya/niok torkekuno?

WHEN PREGNANT/ UKIWA MJAMZITO/ ENIAKU ENDUA

1 What do you eat/drink in the wet season, when you're pregnant?

• Unakula nini?/kunywa wakati wa mvua, ukiwa mjamzito?

• kainyoo oshi inya/iok te niaku endua?

2 How much do you eat/drink each day?

• Unakula kiasi gani/kunywa kila siku?

• ke baa en daa ninya/niok torkekuno?

3 What do you eat/drink in the dry season, when you're pregnant?-ukiwa mjamzito?

• Unakula nini?/kunywa wakati wa ukame, ukiwa mjamzito?

• kainyoo inya/iyok to lamei te niaku endua?

4 How much do you eat/drink each day?

• Unakula kiasi gani/kunywa kila siku?

• ke baa en daa ninya/niok torkekuno?

5 Do you eat/drink the same foods during all of pregnancy?

• Unakula/kunywa chakula ya aina majo kwa mimba yote?

• ke n daikin naanyanyuk oshi inya/iok te niaku endua?

6 Do you eat/drink the same amount during all of pregnancy?

• Unakula/kunywa kiasi iliyofanana kwa mimba yote?

• ke risio en daa ninya/niok te tuaishu ino pookin?

POSTNATAL/ BAADA YA KUJIFUNGUA/ ENDOMONONI

1 How much do you eat/drink each day?

• Unakula/kunywa nini kila siku?

• kainyoo inya/niok torkekuno?

2 How much do you eat/drink each day?

• Unakula/kunywa kiasi gani kwa siku?

• ke baa en daa ninya/niok torkekuno?


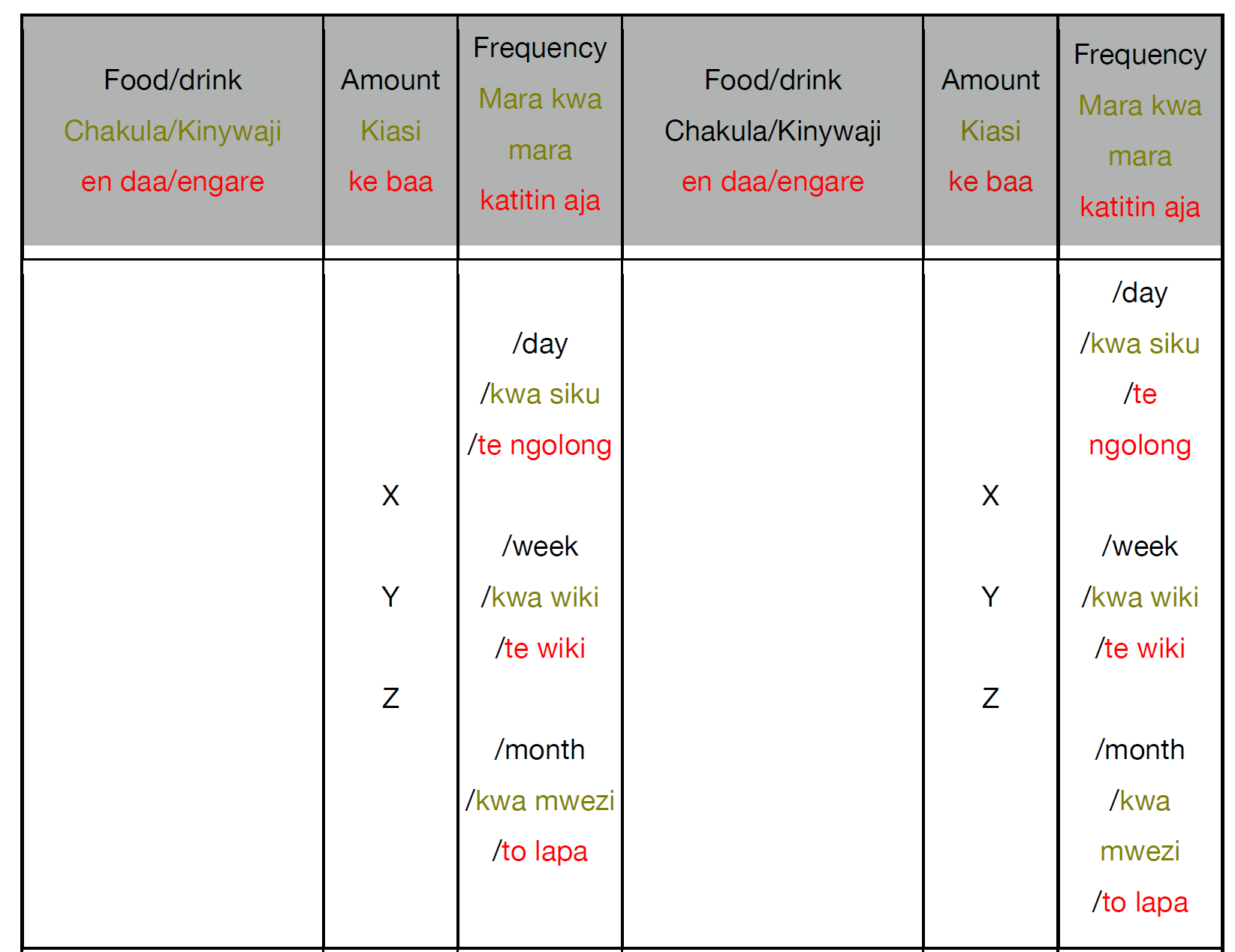


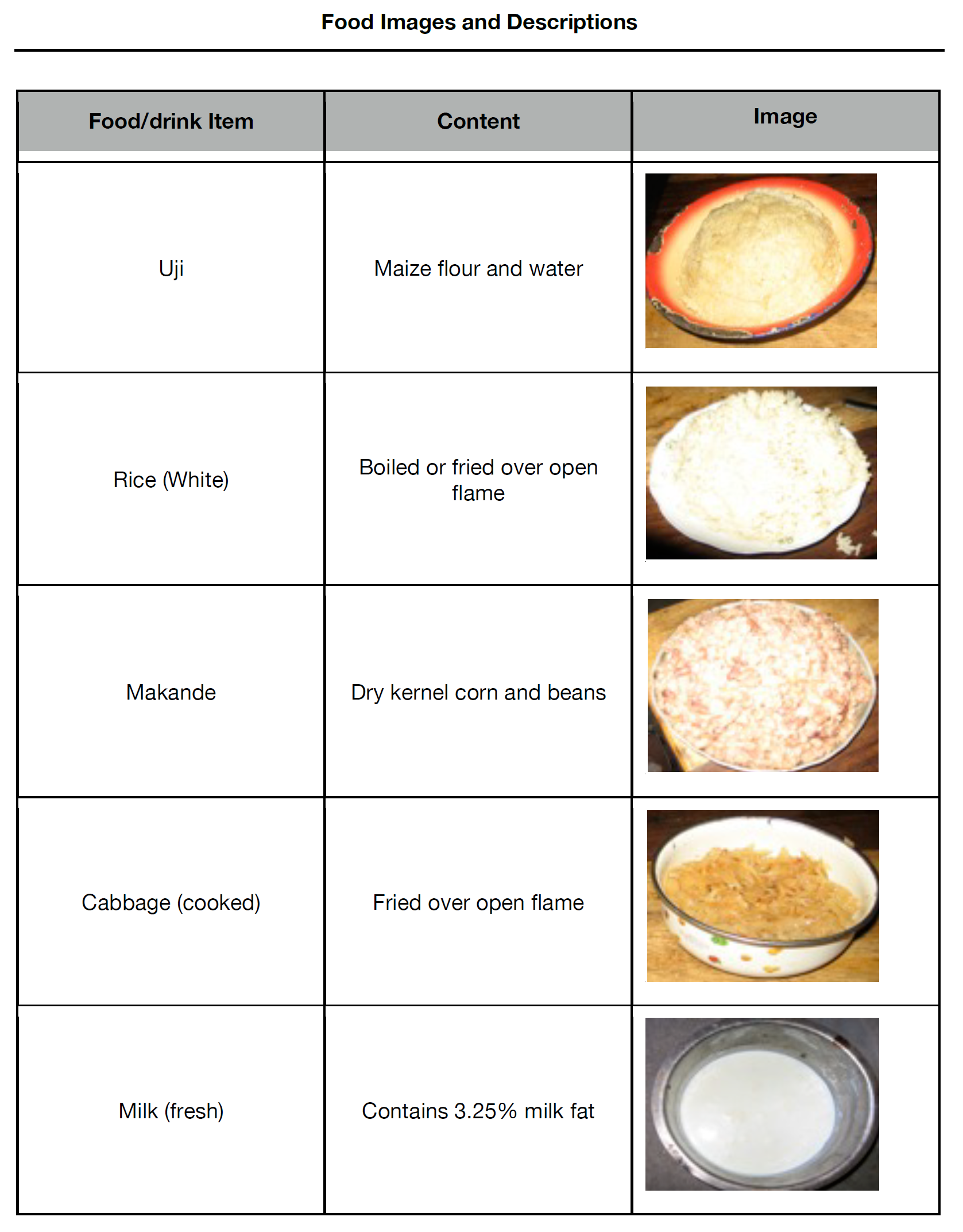


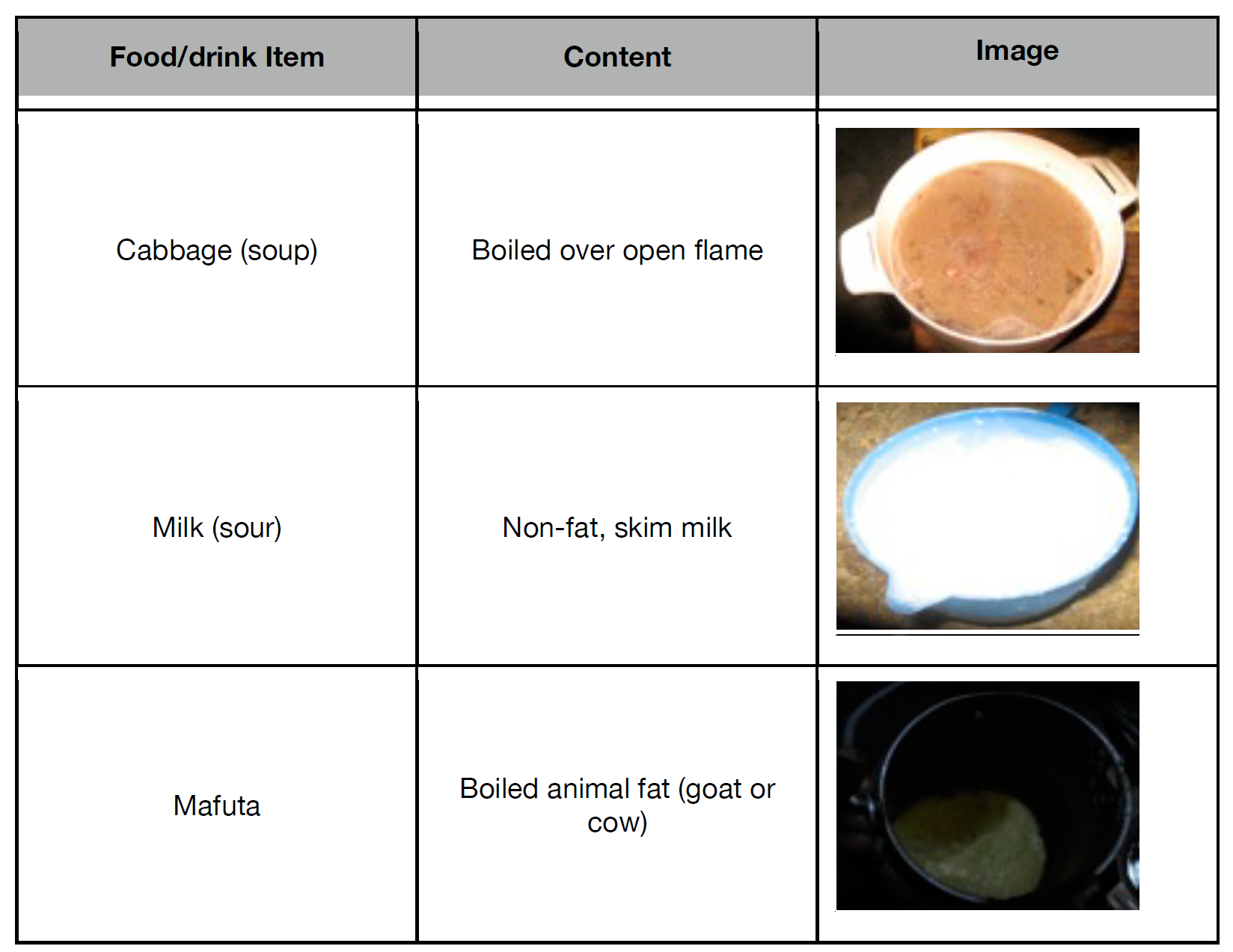


ANTHROPOMETRIC MEASUREMENTS


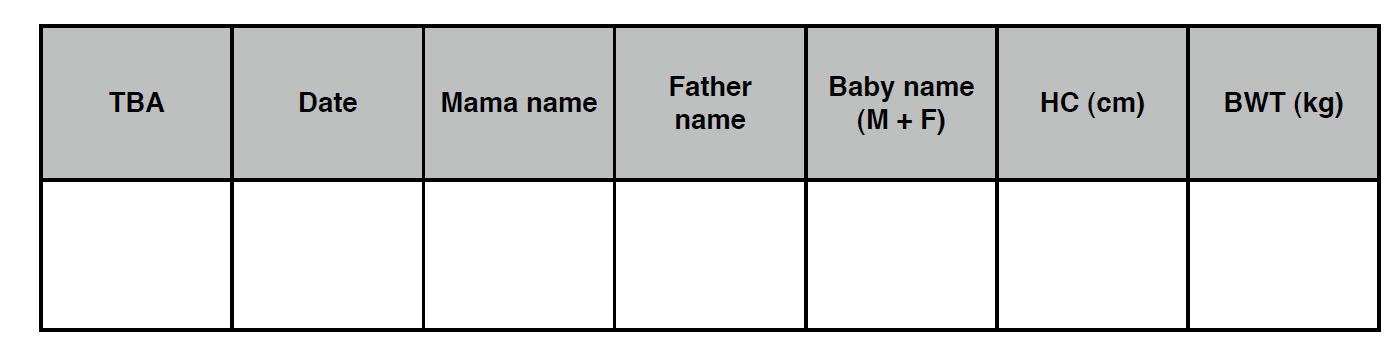


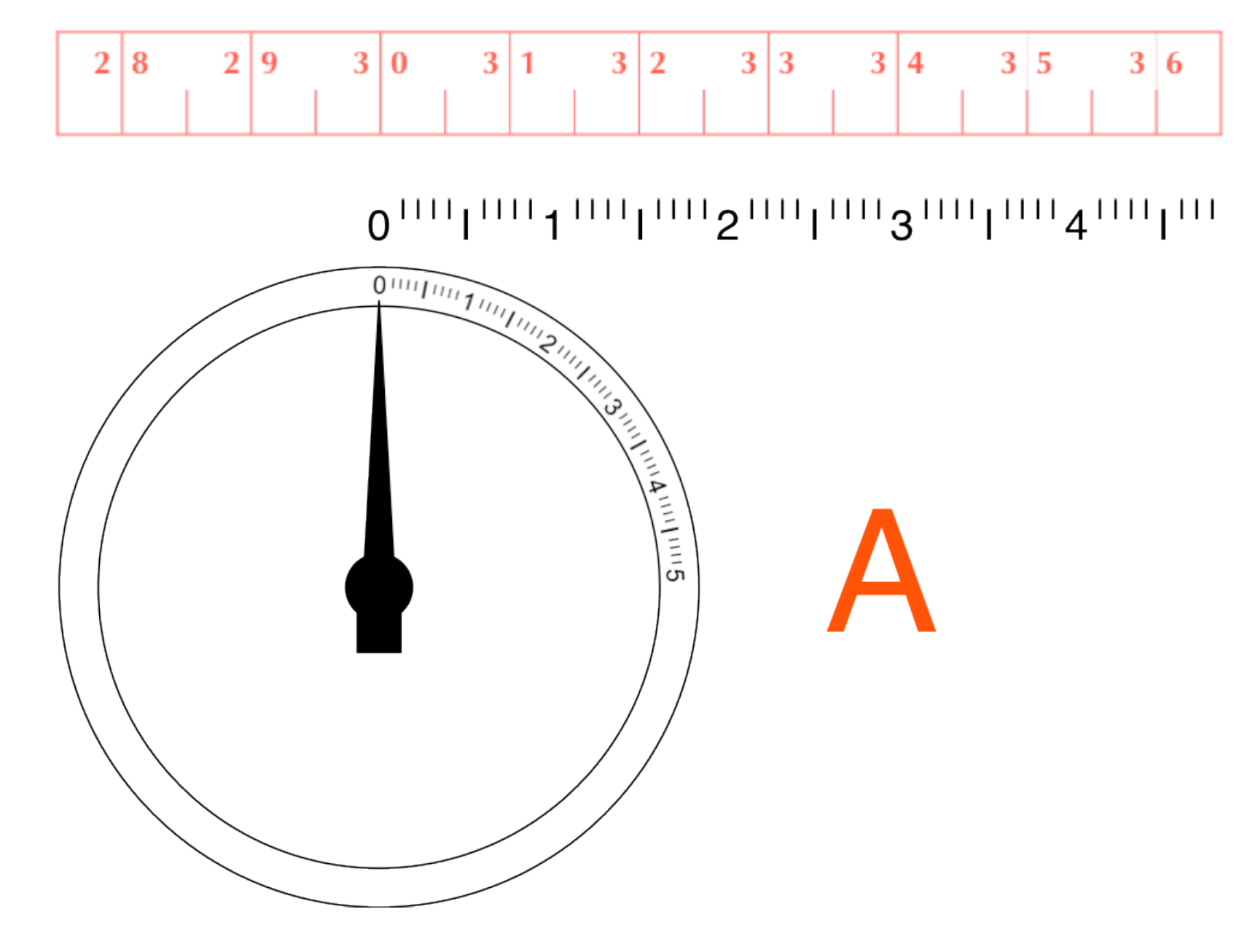

Supplement: S1 Appendix — (DOCX) [file pone.0237700.s004.docx]
