# Supplementary material for: Lack of head sparing following third-trimester caloric restriction among Tanzanian Maasai
Source: PLoS One. 2020 Sep 23;15(9):e0237700. doi: 10.1371/journal.pone.0237700 (PMC7510984; doi:10.1371/journal.pone.0237700)
Supplement: S2 Appendix — (DOCX) [file pone.0237700.s005.docx]

**S2 Appendix. Summary of the main statements emerged in the interviews.**

***Theme 1: Reasons for restricting diet.*** During the first two trimesters (T1-2), diet remained the same as it was prior to pregnancy. At the beginning of third trimester (T3), “dangerous” foods (e.g., fresh milk, moderate cooked meat) were replaced by “safe” foods (e.g., sour milk, animal fat), and intake quantities were significantly reduced. Although absolute quantities of all macronutrients were restricted, fat and protein content was augmented relative to carbohydrate content. These dietary changes were intended to limit fetal growth and mitigate difficult labour and delivery. The following statements were made by three participants during one of the group interviews about dietary practice:

*Women are encouraged to eat much less than they normally eat. This is to prevent her from getting fat, and to keep the baby small.*

*When the baby is coming, it is hard for the mama. We try to make an easier birth by keeping the baby small.*

*If the mama does not eat much food when she is pregnant, then she will not get fat. If she does not get fat, then the baby will be small.*

In addition to limiting fetal growth, women restricted their diet while intending to keep their bodies “clean”. According to this perspective, a clean body is one that readily absorbs nutrients contained in the postnatal diet: “*This is so at birth, the [infant] body will be clean and it will absorb all the food.”* If Maasai women do not restrict their maternal diets, they tend to be criticized by other women for risking complicated childbirth:

*We know a woman who is eating whatever she wants. She is getting fat, and so she will have a big baby.*

***Theme 2: Use of Indigenous Medicine.*** TBAs advise pregnant women to ingest *La Maa* (i.e., indigenous medicines) with emetic properties. Third trimester vomiting is a common maternal practice, and is presumed to constrain fetal growth by limiting nutrient uptake by the fetus.

*When she [pregnant women] is almost at the end of her pregnancy [~ 7 months], she will take medicine to make her vomit. If she vomits, then the baby cannot use the food she eats to get bigger.*

The toxic characteristics of *La Maa* were acknowledged during interviews, and TBAs discourage its use *“until later in the pregnancy [when] the baby is able to grow healthy.”* One of the TBAs specified *“If [I] give to the mama too early in the pregnancy [earlier than 4 months], they [indigenous medicine] might slow [fetal] growth, and cause abortion.”*

Indigenous medicine includes *Osokonoi*, *Olmukutan* and *Endokushi*. Osokonoi is derived from the inner bark of the osokonoi tree (sp. *Warbugia ugandensis*). Approximately one tablespoon of inner bark was shaved and mixed with 125 ml of water. This bitter tasting solution is consumed early in the morning, twice per week. Informants reported that the emetic properties of osokonoi solution are thought to further restrict fetal growth. Because TBAs consider the fetus to be a scaled-down version of a full-term infant, fetal ingestion of nutrients is presumed to happen orally in the uterus. Hence, the acerbic taste of osokonoi is believed to “condition” the fetus to be reluctant to consume nutrients derived from the maternal diet:

*Osokonoi is bitter tasting, and because the fetus takes food through the mouth, it will not like the taste [of osokonoi] and it will think that all food will be bitter tasting.*

*Olmukutan* is procured from the root of the *olmukutan* tree (sp. *Albizia anthelmintica*). Approximately 15 cm long and 5 cm wide root segments are soaked in warm water, and subsequently left to stand until opaque and slightly gaseous. The *olmukutan* solution is filtered and consumed early in the morning, 3-4 times per week.

*Endokushi* was previously used for the similar purposes as osokonoi and olmukatun, but is now avoided because it is widely known to be toxic and to promote miscarriages.

*A long time ago, this medicine [Endokushi] was given at the beginning of pregnancy, but many women got sick and sometimes the baby would come early and die.*

*We used to give it [Endokushi] to help with the pregnancy, but we don’t use it anymore. It is lethal to the baby.*

During interviews the botanical source of endokushi was not disclosed. However, TBAs retain samples of endokushi and impart their knowledge to novice TBAs about its toxicity to prevent its reinstatment.

***Theme 3: Domestic workload and physical exertion.*** Maasai women do not reduce their domestic workloads during pregnancy. Workload appears to decline at the beginning of the first trimester because “*you are tired”*, but steadily increases over the initial four months of gestation while women accumulate firewood, water, and maize flour in preparation for postnatal recovery. During the three months following childbirth, new mothers remain in their homes to recover from pregnancy and care for their infants. One of the participants explained:

“*Some women will even give birth on the way while collecting firewood because men don’t collect firewood, so you will be cold and cannot cook. It is up to you to prepare.”*

*“Just until before the baby comes she will do more work than she does when she is not pregnant because she must collect enough extra firewood and water to let her stay in the boma and be with only with her baby.”*

Participants reported that intense workloads are essential to facilitating the birthing process because *“the extra work helps to make the birth process easier; it helps the baby come”*.
