# Supplementary material for: Lack of head sparing following third-trimester caloric restriction among Tanzanian Maasai
Source: PLoS One. 2020 Sep 23;15(9):e0237700. doi: 10.1371/journal.pone.0237700 (PMC7510984; doi:10.1371/journal.pone.0237700)
Supplement: Attachment — Submitted filename: Powell et al Reply Sept2019.docx [file pone.0237700.s006.docx]

Ref: PMEDICINE-S-19-02168. Response to reviewer´s comments

Dear Editor,

Please find enclosed our revised version of the manuscript entitled **“Dietary practices during pregnancy among Tanzanian Maasai and its effect on growth status at birth”** by Powell et al., which we previously submitted to Plos Medicine.

We thank the reviewers for their comments which help to improve our manuscript. We provide a detailed description of the changes introduced according to reviewer´s suggestions.

Sincerely,

Reviewer #1: I have read the manuscript Dietary Practices during pregnancy among Tanzanian Maasai and its effect on growth status at birth, with much interest, and authors are to be commended for such original research. The manuscript is well written and the results support the conclusions. The pregnant women from NCA showed a dramatic decrease in dietary intake in the 3rd trimester, resulting in lower birthweight and particularly also in lower head circumference at birth. It seems very convincing that this intake reduction is the main cause, but while the authors do address some alternative explanations in their discussion, I do have some further issues to consider.

- The participating women were described using emetics in the third trimester in order to help reduce food intake. Is there any information on the substance used and whether this could have caused, or contributed to, fetal growth retardation?

**Response:** We added the name of two species with known emetic properties. As they were used in combination with diet restriction is hard to assess their specific contribution, although as we point out in the manuscript they might have an additional effect on fetal weight loss.

- Authors write that there is seasonal food insecurity, and shortages in the dry season of water and food. It is noted on page 11 that all participating women gave birth in the dry season, so for many of the women the third trimester was also in the dry season. Could this have contributed to lower food intake?

**Response:** The reviewer raises an interesting point. As we discuss later, the third trimester restriction occurs on a background of food shortage, so the concurrent effect of these factor can not be completely ruled out. It would be very interesting to compare the baby outcome in pregnancies at different times of the year, however; the fieldwork took place during the dry season because several areas can not be reached during the wet season.

- In the Discussion, p26, it is explained that also in trimesters 1 and 2 the food intake of participants is far below recommended intake by FAO. Authors state that the lack of evidence for head sparing is remarkable, but as they write on p27, given this very low intake to begin with, amplified by further restriction in the third trimester this effect may not be so unexpected. Do authors exclusively attribute this lack of head sparing in these women to the intake reduction in the third trimester (p27, l 506) or do they consider it a result of low intake over all pregnancy, including severe reduction in the third trimester?

**Response:** As we discussed in the manuscript, our study shows a significant reduction of food intake during the third trimester, which occurs on a background of food shortage. Clearly, the phenotype of newborns is influenced by the maternal environment experienced throughout gestation. The point we make here is that under a situation of maternal undernutrition we expected a more significant reduction of birth weight than HC, which is not observed for this population.

- On page 13 lines 250-252 it is stated that authors were aware of (and tried to prevent) socially abberant behavior. Would that social pressure mean that reducing food intake in the third trimester is the socially acceptable action? Maybe add some explanation about this?

**Response**: We modified this sentence as: “We recognized that participants’ responses may have been influenced by social desirability, and that participants may have been reluctant to disclose their actual dietary habits to avoid criticism if they do not conform the socially encourage practice of reducing food intake during the third trimester. To mitigate this potential bias, open-ended questions and informal dialogue to encourage active conversation were combined with participant-observation to confirm interview content.”

- On page 14 line 280 it is explained that FFQs were taken from participants by TBAs shortly after delivery. Given that social pressure for desirable answers might play a role, were individual TBAs taking the questionnaire from individual participants?

**Response**: Questionnaires were made by individual TBAs to individual participants. We clarified this in the text.

- Were anthropometric data (page 16, top) gathered by TBAs or perhaps other care workers? With respect to the possibility of information bias, if it were TBAs who did these measurements, did they first take these outcome measurements and thereafter the dietary measurements?

**Response**: Anthropometric data were gathered by TBAs trained by one of us (CP). To facilitate the measurement procedure and reduce the observer error, we provided images of the scale-face and paper tape in the booklet so that numeric values could be circled instead of hand-printed. We added this explanation to the new version of the manuscript.

- In the analysis of nutrition (determinant) data, systematic measurement error by TBAs was already accounted for. In 'Data analysis of anthropometric variables' (page 16), the anthropometric (outcome) data was standardized for TBA effects. In the final analysis of outcome (HC/BW) as a function of the determinant (calorie intake), again TBA was set as a random factor. Was the anthropometric outcome in such modelling the TBA adjusted values?

**Response**: We changed some of the analysis and the mixed models testing the outcome as a function of calorie intake were removed.

- On page 16, it is stated that a 4 sd cut off was used for outlier classification. It seems somewhat odd to use a rather arbitrary cut off and then exclude such values when those may just be extreme (true) values occurring in almost all such measurement distributions. Were these outliers just erroneous or extreme? Could authors add some more information on analyses that included such outliers?

**Response**: The 4sd cut off was used on the basis of recommendations for the treatment of outliers in the literature (please, see reference in the manuscript) as well as from growth studies in which measurements greater than -4SD are excluded as they might be related to factors other than growth restriction (e.g. congenital anomalies).

Reviewer #2: Generally an interestING paper with potential to contribute to this unique evidentiary gap. I am at times concerned with the over-statement or lack of tentativeness in your writing and would strongly suggest a re-read and editing with this in mind. Your reporting is interesting although there seems to be an under-reporting of the qualitative aspects. As well you likely could do more work on integrating the findings of qualitative and quantitative findings from a strengths based approach. However, this latter suggestion is perhaps outside the requirements for this submission, but more a future consideration in maximizing your mixed methods data.

**Response**: The suggestions of the reviewer are interesting and we agree that qualitative data are a relevant aspect that needs a deeper analysis. However, it should be noted that a qualitative analysis of the data is beyond the aims of this study. We plan to do so in a future study.

216-221 I am confused as to the actual representativeness - this is a very small portion of the NCA Maasai - you would only have reached as small catchment - or could you please provide the actual geographic spread of the individuals - the lack of demographics/descriptives of the sample is disconcerting as there are variations in food and accessibility across the crater. Use patterns to Endulen hospital also vary across the crater. Also the TBAs are very 'regionally' or 'locally' based and would not reach beyond a small service area. Are all 12 from the Endulen area - for example how many are from Olbalbal or Misigyo or Alelaylai? I would appreciate a better sense of the involvement and representativeness of the sample. Another issue in your sampling is whether you captured data such as parity, income, education, etc. Were there variabilities seen in the dietary practices? Our experiences would suggest that more educated and multiparous women were less likely to adhere to the rigorous dietary customs. Do you have further considerations of this potential implication?

**Response:** All of the TBAs are from the area around Endulen. The meetings with the TBAs as well as all of the training for anthropometrics were performed at the Endulen hospital. All TBAs are drawn from the roughly 10 km radius around Endulen. So, the reviewer’s point is an excellent one. This sample is not necessarily representative of the entire NCA are and we have added a statement to that effect. We have also added a brief description of the ecological setting of the sample. Unfortunately, we do not have Information about income level, although our sense is that it is fairly homogenous. This would be a difficult variable to assess given the social setting. Unfortunately, we do not have parity data for the participating mothers. The reviewer is correct that this may influence adherence to the food practices. While this would have been a valuable variable to have, it does not directly affect the outcomes that we report in this paper. Parity Information will be included in future work.

248-256 This section was confusing as you indicate that the interviews were held with the TBAs but then you speak about participants - but this reporting seems to be about the women (pregnant) not the TBAs. Hence this needs a reconsideration for clarification and intent.

**Response**: We clarified this section.

254-255 who was involved in the initial group interview - with what intent? Was that data included in the thematic content consideration?

**Response**: To investigate maternal food intake throughout gestation, one of us (CP) first conducted a series of group interviews and generated observational field notes. On the basis of these notes, we performed the questionnaires that were then used for collecting information on food intake.

293/4	I am confused that you asked about T1-2 diets as well given this is now getting to be a historical recall (beyond 90 days).

**Response**: The reviewer is right, we asked about the diet during the first two trimesters. As we mention in the text given that the maternal diet is not altered until five to six months gestation, recall for early to mid-gestational diet was no longer than 3-4 months, which is an acceptable window for recall when using the FFQ method. Moreover, the NCA diet is monotonous, which lends to reproducibility and accurate recall because fewer types of food items are consumed on a regular basis.

306	To confirm these measures were done in the home? Or done in hospital? It is confusing when most Maasai women do not attend at a hospital for delivery and seldom for the post-partum follow up. So are the women you are assessing all using hospital delivery only? Again this is an issue of your sample description.

**Response:** The measurements were taken by the TBAs in Boma in which each birth took place.

340-348 - it would have been helpful to have a direct quotation or two to emphasize the key points and to attribute to the women who were your participants.

**Response**: We included a summary of the main statements that emerged in the interviews in S2 Appendix.

462 what is meant by 'further attenuated caloric intake to 799 kcal/day) - something is missing in this sentence respecting T3

**Response**: We added the reference to T3 in the sentence.

464-467 this needs to be re-written for clarity

**Response**: We re-wrote the sentence as “United Nations Food and Agriculture Organization and the World Health Organization [6] recommend that macronutrient energy consumed by adults should consist of 55-75% carbohydrates, 15-30% fats, and 10-15% proteins.”

487-489 so were your Bugando people Maasai or primarily Sukuma? There are tribal lines and genetics that might impact - please revisit.

**Response**: As the reviewer notes, Bugando people was primarily Sukuma. These differences in genetic background between samples can not be ruled out, although several studies show that environment rather than genetics is the major determinant of birth weight and HC. We included this discussion in the new version of the manuscript.

529 what is meant by the 'noisy nature of the food survey data' ?

**Response**: We modified the sentence as “The limitations of the food survey data mentioned above make the significant values and the consistent reduction of food intake across TBAs all the more remarkable. “

Reviewer #3: I confine my remarks to statistical aspects of this paper. The general approach is fine, but I have some comments that will add clarity to the manuscript.

One fairly large addition that might be useful is to look at the reduction in calories, rather than just 3rd trimester calories. Or to look at a multilevel model (aka mixed model) with the calorie intake for each trimester. This could also be graphed in what is known as a spaghetti plot.

**Response**: We performed a linear model to assess the reduction in calories between the first two and the third trimester. Results are shown in Table 1.

Line 105 ff What are the numbers after the +- sign? Is that a standard deviation? A 95% CI? or what? In either case, I think the whole number is fine. Fractions of a calorie aren't really important.

**Response**: We incorporated the suggestion of the reviewer

I would use grams and cm a lot more, rather than z scores - or give both where the z score is important. Z scores are abstract. We have intuition about what 100 g of birthweight is, but not what 0.5z is.

**Response**: We reported both, the raw measurements and z/scores. The z scores are the standard in growth studies, so we reported them for the sake of comparison with other studies.

Lines 318-325 Please give a bit more detail. In the ANOVA, what was the DV and what were the IVs? How does standardizing to the grand mean remove bias? Looking at the subscripts of the F, it appears that the IV was the individual TBA (since there were 12 of them) but this should be stated. I am pretty sure that what was done is correct, but it could be clearer.

**Response**: We incorporated the suggestions of the reviewer

Line 332-334 Why not explore with a spline of third semester calorie intake? I am not an expert in the substantive area, but, intuitively, I think it reasonable to wonder if the effect was linear.

**Response:** We are not sure we understand this suggestion of the reviewer.

Figure 2: Rather than these plots (known as "dynamite plots") it would be much better to show a box plot, perhaps with individual points overlaid, like the top part of Fig. 3.

**Response**: We replaced the plots by box plot as the reviewer suggested.

Table 1 This is not the right table for displaying a linear mixed model. Why not use a more typical format, with columns for beta, SE, p and a CI?

**Response**: We modified the table accordingly.

Table 2: The column labels in "Sample" should be vertically centered between the 2 lines in each half of the table. This is another place where the value after the +- sign needs to be specified. Also, given that the medians are very close to the means, it's not clear that they need to be included. It isn't wrong to do so, but why are they there?

**Response**: We incorporated the suggestions of the reviewer.

I would consider adding regressions with HC and BW as DVs and the sample, the baby's sex and the interaction.

**Response:** The regression lines for HC on BW by sex are shown in Figure 5.

Figure 3: Here I would definitely use grams and cm, not z scores. In the bottom panel, the lines for M and F need to be differentiated, e.g by using a dashed line for one and a dotted line for the other. (Also for Fig. 4, left panel).

**Response**: We differentiated the lines for M and F. With regards to the use of raw measurements and z scores, please see the comment above.

The figure for TBAs would be enhanced by adding dots for each case.

**Response**: We incorporated the suggestion of the reviewer.

Despite all the above comments, this is a good (and disturbing) paper.
