# Supplementary material for: Lack of head sparing following third-trimester caloric restriction among Tanzanian Maasai
Source: PLoS One. 2020 Sep 23;15(9):e0237700. doi: 10.1371/journal.pone.0237700 (PMC7510984; doi:10.1371/journal.pone.0237700)
Supplement: Attachment — Submitted filename: Rebuttal letter Jan2020.docx [file pone.0237700.s007.docx]

Reply to Reviewers and Editor comments

**Ref. Ms “Dietary practices during pregnancy among Tanzanian Maasai and its effect on growth status at birth”**

Editor Comments

**Comment** 1: A food frequency questionnaire (FFQ) was developed and used. The easiest
way to get food intake from each trimester would be a few 24 h recall. Why authors choose the FFQ?

**Reply:** The different methods for dietary assessment proposed in the literature all have strengths and limitations (see review in Shim et al., 2014, Epidemiology and Health, 36). The selection of one specific method will depend on the aims of the study and the feasibility according to the human and material resources available. The 24h recall is a subjective measure based on open-ended questionnaires administered by a trained interviewer. Its main adjvantage is that it provides detailed intake data and no literacy is required. One limitation for the purpose of our study is that the method mainly focuses on short-term intake, while we were interested in long-term dietary exposure. This means that multiple interviews would be needed to assess food intake. This is both an expensive and time-consuming. Based on our resources this was deemed to be inadequate for our study in Tanzania. Additionally, a possible negative consequence of this method is that it can result in changes to diet as a result of repeated interviews.

On the other hand, the FFQ method estimates food intake over relatively longer periods. It is both cost-effective and time-saving, and thus suitable for epidemiological studies (Jackson et al., 2012, Public Health Nutrition: 16; Zack et al., 2018, Public Health Nutr. 21). Since the FFQ is specific to each study group; we carrefully designed and validated the questionnaire before gathering data on dietary intake among Maasai women.

Briefly, a group interview was first held with a group of traditional birth attendants and mothers. Participants represented a cross-section of ages (30-50 years), were multiparous and were familiar with dietary restriction. The initial group interview focused on the rationale for structuring and implementing dietary survey. Then, a workshop was held with the TBAs, who were asked to list food items that are commonly consumed during pregnancy. These items were recorded and compared to previous studies on maternal diets of Kenya Maasai (Nestel 1989; Mpoke 1993). Upon confirming the relevance of our initial food list, we developed a first draft of the FFQ which was disseminated to the TBA group interview participants, and was reviewed by randomly selected women at various stages of pregnancy (N=36). These women confirmed the relevance of the initial food item list, and suggested additional items for the FFQ, which was distributed to the TBA field team. The TBAs had occasion to practice interview, and provided feedback about how to improve data accuracy. Then, we held a training session to review data collection techniques and the TBAs proceeded to the data collection phase.

**Comment 2:** It is not clear in the text the frame time for the FFQ neither how many and when the FFQ was obtained. The text reads 2-3 days postpartum and a time frame of 3-4 months?

**Reply**: Maternal data were collected two to three days postpartum. During the interview, the TBAs asked about the dietary intake during the first two trimesters and the last trimester, filling out a FFQ for each period.

Since maternal diet is not altered until five to six months into pregnancy, recall for early to mid pregnancy was no longer than three to four months, which is an acceptable window for recall when using the FFQ method (Buzzard et al., 2001; Michels et al., 2005). Moreover, because of seasonal food scarcity and limited subsistence options, the NCA diet is monotonous, which lends to reproducibility and accurate recall because fewer types of food items are consumed on a regular basis (Beaton et al., 1979; Kigutha, 1997). A total of 68 FFQs for the first two trimesters and 79 for the third trimester were included in this study. We incorporated this information in the Results section of the new version of the manuscript.

**Comment 3:I**n results “The diet of Maasai women during T1-2 remained the same as it was prior to pregnancy”, but FFQ was reported for pre-pregnancy? It is quite confuse the points in time of FFQ. In order to compare trimesters, dietary intake during trimesters one and two were combined? How the mean? This is not appropriated. Changes over the three trimesters of dietary intake would be the adequate approach.

**Reply:** In the reply to the first comment we provided more details about the methods for developing the food frequency questionnaire. Before implementing the FFQ, one of us (CP) had group interviews with TBAs and mothers in which they were asked about their dietary habits. From these interviews it was established that dietary changes occur later in pregnancy. This is in agreement with findings for other Maasai groups. On this basis, we decided to focus the FFQ on dietary intake during two clearly differentiable periods of pregnancy, before and after the pregnant women start the dietary restriction. We agree with the reviewer that it would be ideal to gather data throughout gestation but this was not feasible because of our limited resources and the difficulties for access both to the study area and the participants. Finally, as mentioned above the diet is very monotonous.

**Comment 4**: How many Infants had no sex information? The outliers plus no sex information is about 20% a large amount.

**Reply**: The sample of Maasai infants with anthropometric data was composed by 140 individuals, from which 24 had no sex information. Consequently, the estimation of z-scores and further analyses based on these scores were performed using the sample of 116 infants with sex information.

**Comment** 5: It is not clear the need to compare the expected HC given the BW for the infants from the two Tanzanian samples based of the values derived from the WHO reference. Comparison between the two samples would be easily interpreted.

**Reply:** The reviewer is correct that a comparison between the two simples can easily be interpreted. However, the analysis as a whole uses the WHO reference as the baseline comparison. It is, therefore, appropriate to determine deviataion from the expected relationship between head circumference and body mass using this reference and to determine how the two samples lie with respect to the WHO sample. There is a statistical advantage to this as the WHO sample is so large that the relationship can be assumed to be measured without error. Further, the point of the comparison to the Bugando sample is simply to show that we do not obtain similar results in another Tanzanian sample in which we have no evidence of third trimester food restriction. It is not to use the Bugando sample as an appropriate baseline comparison. Had we done the comparison as the reviewer suggests, our study could be criticized on the basis of the appropriateness of the Bugando sample as an “optimal nutrition baseline” which it is clearly not.

**Comment 7**: Fig 1 and 2 could be moved to supplementary data.
**Reply**: Figure 1 was moved to supplementary data, while figure 2 was eliminated from the new version of the manuscript.

**Comment 8**: In figure 3- If T1 and T2 were averaged we expected less variation compared to T3.

**Reply**: The larger variation for the first period suggests the existence of individual differences in the food intake which are reduced by late gestation, when dietary intake is more regulated by cultural practices. Given this, it is not unexpected to find more variation for T1-T2. Also, it should be noted that, as we explain above, information about food composition for early-middle pregnancy was not obtained separately for each trimester. Consequently, the values in Figure 3 do not represent an average.

**Comment 9**: Fig 5- authors could keep only the figure with the curves, data on the right is less informative.
**Reply:** These figures both contain Information necessary to understand the paper. We have addressed this by better explaining these figures in the text and captions.

Reviewers' comments:

Reviewer #1: Comments to the author

**Comment 1:** Was the food consumption of the Bugando sample not assessed? How to ensure that there was no decrease in the food consumption of some mothers, given the variety in the socioeconomic context, as mentioned in the text? I consider that it is not sufficient to state that “there is no evidence of specific third-trimester food restriction in that population”.

**Reply:** The Bugando sample was incorporated to compare the anthropometric measurements at birth with a Tanzanian population in which the cultural practice of third trimester restriction is not extended. The women included in this sample attended antenatal clinics during the pregnancy period to monitor the progress of the pregnancy. During the antenatal visits, they were given health education regarding nutrition. The information provided by healthcare practitioners does not report any specific diet restrictions among these pregnant women, although food consumption was not assessed in Bugando sample because it was not part of the original research project. Therefore, even though we can not discard variation in food intake due to socio-economic status and availability of food, we consider this is an appropriate reference for comparative purposes.

**Comment 2:**- Line 347 – “it was after accounting for TBA and maternal effects” What factors were these considered? The title of table 1 also needs to be improved and incorporated into the footer which are the adjustment variables.

**Reply**: We modified the title and provided more information about the variables used in these models.

**Comment 3:** I suggest further analysis on association between reduced food intake (difference between T1-2 and T3 - continuous or tertile) on newborn size, controlled for "TBA and maternal effects”. Perhaps this association could reinforce causality by showing a dose-response relationship.

**Reply:** Following reviewer’s suggestion, we estimated linear models with weight and HC as dependent variables, the amount of reduction in diet components (calories, protein, carbohydrate, fat) as fixed effects, and TBA and mother as randon effects. We found a significant relationship between infant weight and calorie and protein reduction, while the effect was non significant for HC. These results need to be taken with caution. Because all Masaai women reduced their food intake, the range of variation of the differences between T1-2 and T3 is relatively small, and thus even if a dose-response relationship is present it would require a larger sample size to be detected. The results from this analysis were included in a table as Supporting Information (S5 Table).

Comment 4: In general, I suggest that the titles of the figures and tables be revised. The information of who and when should be added.

Table 2: after the +/- sign would be the SD? The information should be included in the header line of the table. Also, information about female (F) and male (M) should be included in the table title.

**Reply**: The titles of the tables and figures were revised according to the reviewer’s suggestion.
