# Supplementary material for: Lack of head sparing following third-trimester caloric restriction among Tanzanian Maasai
Source: PLoS One. 2020 Sep 23;15(9):e0237700. doi: 10.1371/journal.pone.0237700 (PMC7510984; doi:10.1371/journal.pone.0237700)
Supplement: Attachment — Submitted filename: Guide to revisions.docx [file pone.0237700.s008.docx]

**Guide to Revisions, PONE-D-19-25660R1**

**Overview: We are grateful to the reviewers for their careful reading of our paper. We agree with many points raised and have used those to improve the paper. Where issues of clarification are required we have modified the text and explained our position below. Below is a point by point description of how we have addressed the reviews.**

Reviewer #2: There are significant editorial issues remaining - ranging from grammatical to spelling. I would strongly suggest that you take time to have the document propoerly edited. Spacing errors and puncttuation errors must be atteded to. Please use the same font size throughout the document (including the reference list).

**Response: We agree completely with the reviewer on this and apologize for not having noticed this issue earlier. This paper has gone through many revisions and several authors have edited and re-edited the paper. This is not an excuse but it does explain why the last version got away from us the way that it did. We have thoroughly edited the paper for language and logical flow. This means that it is not really possible to highlight specific bits of text that are changed as virtually the entire paper has been intensively edited. We thank the reviewer for pointing this out.**

Reviewer comment: I struggled with your comparison to the Bugando maternal-child dyads - these are non-Maasi peoples with very distinct 'tribal' variances that you have not corrected for. I am not convinced you actually can make this a reasonable comparison. Even the urban/per-urban factor was a further confusion, as the NCA residents are rural pastoralists. i remained confused as to why you did this component of the study given the minimal data collected.

**Response: Yes, this is a good point. We included the Bugando sample simply because as we were conducting our study at Endulen, we were struck by the paucity of comparative data from African populations. That remains a problem. We did not intend this cohort to serve as a control sample because there are obviously many factors that distinguish these groups. Our primary comparison is to the WHO reference data. We do feel that inclusion of the Bugando data has value, if only to illustrate how strikingly the Endulen newborns deviate from the WHO norms. An ideal comparison might have been Maasai newborns from a community in which the third trimester dietary restriction was not practiced. We did try to identify such a community in Tanzania but were unable to identify one. The issue with the appropriate control remains and it is now discussed as a caveat in the discussion. We hope that this is acceptable as we believe that the findings are sufficiently striking to overcome this limitation in the research design of our study.**

Reviewer comment: your consnet process also remains questionable. I would want more details about the TBAs role as my knowledge of this population (where I have done many studies) would indicate a concern with the TBAs literacy. So if you are asking them to read the consents to the participants this is highly unlikely from my experience. I would stress that you explain what was actually done.

**Response: We have added text to clarify the precise role that the TBAs played in ours study design. Relying on the TBAs for the consent process was an issue that was extensively discussed with CHREB (the UCalgary) IRB at the time of study design and with NIMR. This was a significant issue in launching the study as we could not implement normal consenting processes for precisely the reasons that the reviewer identifies. We also certainly understand the reviewer’s concern as this was an issue that we discussed at length. Given the logistical and, more importantly, cultural challenges involved (which we suspect that the reviewer understands, based on the comments), there was really no other way to recruit the subjects and obtain the anthropometric data required for this project than by training and relying on the TBAs. In the end, the system that we implemented relied on verbal consents that were overseen by a local translator (Godwin Olesaningo) who is also a long-standing member of our research team for this and related work. Mr. Olesaningo trained the TBAs in conjuction with Mr. Powell, whose M.Sc. work was based on this research. Mr. Olesaningo served at the time as the community outreach coordinator for Endulen Hospital and his primary contacts for maternal health in the community were the group of TBAs recruited for this study. Paula Gonzalez was also present in the field but only for a portion of the study period. So, this creates the additional complexity that two male researchers, even if one of them is Maasai, would have virtually no direct access to the mothers recruited to this study for reasons particular to Maasai culture. The training of and reliance on the TBAs was the only way that these very sensitive data could be obtained. This means that we have records of the verbal consenting process and training of the TBAs but not for the mothers themselves. We fully realize that this is unusual and that it poses ethical questions about the validity of consent processes in non-literate, closed cultural settings such as the one in which this study occurred. CHREB approved this unusual process only with the provision that it would also be approved by NIMR. NIMR did approve our process as described and we have the documentation for all of this deliberation. While we completely understand the concern of the reviewer, we respectfully ask that they accept the deliberation and decision of the ethics boards that oversaw our work.**

**An advantage of having worked with the TBAs in this way, is that they became much more actively engaged in the work than otherwise. This is important as future interventions in this and other similar communities must leverage the care and expertise that the TBAs provide. This, of course, raises a different ethical question as to why, if the TBAs were involved via the Focus groups and training sessions in the design and conduct of this research, are they not authors on this paper. This is also an issue that we struggle with as our preference would certainly be to include them as authors. However, as the reviewer states, these women, whilst knowledgeable and intellectually capable of engaging with the work on a verbal basis, are not literate. They would not be able to meaningfully engage with the written manuscript. Further, there is content in this paper that is potentially controversial, as it relates to ongoing food insecurity within the NCA which is exacerbated by policies of the NCA itself. This would create risk for these women that is not commensurate with the small benefit of authorship on this paper.**

Reviewer comment: The dietary recoall for 9 months is much more is a high expectations. The dietary intake is only one aspect of the nutrition in this gorup - for example, herbs to cause emesis(vomitting) and what quality of food is actually allocated has not been addressed in your paper. I am concerned about this possible misrepresenation.

**Response: This is an excellent point. We provide caloric estimates based on the FFQ data but we agree that we should be more transparent about the limitations of these data. We have added a discussion of these issues in the Discussion session as caveats for the interpretation of these results.**

Reviewer #3: Interesting and well written report on the association between third trimester maternal food intake restriction and birth sizes. The report is transparent with regard to the methodologies applied and possible alternative explanations, but there are some issues to address further.

Reviewer comment: Authors rightfully discuss social desirability as a possible source of bias with TBAS’s. However, could there also be another source of bias? TBA’s collected maternal dietary data 2-3 days postpartum, presumably having already measured birth sizes. How exactly do socially desirable answers play a role once the child was already born alive and presumably healthy albeit (too) small? A second question is if the knowledge of birth sizes could have influenced measurements through FFQ’s? Or, for that matter, have there been occasions where FFQ’s were taken before birth size measurements, where knowledge of birth sizes may have influenced the actual birth measurements? If this kind of information bias, which cannot be analytically solved, could have occurred it should be briefly discussed in the limitations section.

**Response: This is a good point. We have added a discussion of this issue in lines 484-490. Specifically, we agree that it is possible that a TBA might wish to ‘protect’ a mother under their care and that this might bias the dietary data to be more consistent with an expectation. It is correct that there is ‘local knowledge’ that pregnant women ‘should’ decrease food intake and change the type of food they eat. However, it would take some level of ‘sophistication’ to alter, for example, the carbohydrate component and not some other component. As mentioned in the next section, it is also unclear if the TBA would know that the newborn is ‘small’ according to the WHO. They would really only know how the newborn compared to other newborns in the area. So, we do not think there is a bias in that regard.**

Reviewer comment: Since there is this obstetric dilemma, is anything known about maternal complications/deaths at delivery or thereafter in the Maasai and Bugando women and, if so, were there differences?

**Response: This is an excellent point about which we have also wondered. Unfortunately, there are no data from Endulen on rates of obstetric complication so it is completely unknown whether the dietary reduction strategy actually accomplishes its purported goal. The vast majority of Maasai births are at home and birth complications are not systematically reported in any way, so these data do not exist, unfortunately.**

Minor points

Reviewer comment: Were neonates born at Bugando Medical Centre sampled during the dry season?

**Response: No, these data were collected in December of 2014 and January of 2015. There would not be a similar seasonal fluctuation of food availability in Mwanza as in Endulen, however. See comments about the relevance of this comparison above.**

Reviewer comment: Table 2: give decimals for HC in Bugando males

**Response: This has been fixed.**

Reviewer comment: Some typo’s here and there.

**Response: We have thoroughly edited the paper for grammatical issues and logical flow.**

**e**
